# Antagonistic Pharmacological Interaction between Sirtuin Inhibitor Cambinol and Paclitaxel in Triple-Negative Breast Cancer Cell Lines: An Isobolographic Analysis

**DOI:** 10.3390/ijms23126458

**Published:** 2022-06-09

**Authors:** Anna Wawruszak, Jarogniew Luszczki, Estera Okon, Arkadiusz Czerwonka, Andrzej Stepulak

**Affiliations:** 1 Department of Biochemistry and Molecular Biology, Medical University of Lublin, 20-093 Lublin, Poland; estera.okon@umlub.pl (E.O.); arkadiusz.czerwonka@umlub.pl (A.C.); andrzej.stepulak@umlub.pl (A.S.); 2 Department of Pathophysiology, Medical University of Lublin, 20-090 Lublin, Poland; jarogniew.luszczki@umlub.pl

**Keywords:** breast cancer (BC), histone deacetylase inhibitor (HDI), sirtuin (SIRT), sirtuin inhibitor (SIRTi), cambinol (CAM), paclitaxel (PAX), anti-cancer drugs, combined therapy, isobolography

## Abstract

Breast cancer (BC) is a heterogeneous disease with different intrinsic subtypes. The most aggressive subtype of BC–triple-negative breast cancer (TNBC) is characterized by high heterogeneity and metastasis rate, poor prognosis and lack of therapeutic targets due to the absence of estrogen receptor, progesterone receptor and human epidermal growth factor receptor 2. Targeted therapies have been approved for many other cancers and even other subtypes of BC, but treatment options for TNBC are still mainly limited to chemotherapy. Therefore, new, more effective treatment regimens are needed. Combined chemotherapy with two or more active agents is considered a promising anti-neoplasm tool in order to achieve better therapeutic response and reduce therapy-related adverse effects. The study demonstrated an antagonistic effect commonly used in TNBC therapy cytostatic drug-paclitaxel (PAX) and sirtuin inhibitor: cambinol (CAM) in BT-549, MDA-MB-468 and HCC1937 TNBC cell lines. The type of pharmacological interaction was determined by a precise and rigorous pharmacodynamic method-isobolographic analysis. The cytotoxic and anti-proliferative effects of CAM used alone or combined with PAX were determined utilizing 3-(4,5-dimethylthiazol-2-yl)-2,5-diphenyltetrazolium bromide (MTT) and 5-bromo-2′-deoxyuridine (BrdU) assays, respectively. Induction of apoptosis in TNBC cell lines after PAX and CAM treatment applied individually or in combination was determined by flow cytometry (FACS) as a number of cells with active caspase-3. It has been observed that both agents used separately inhibit cell proliferation and induce apoptosis; however, applying them in combination ameliorated antiproliferative and pro-apoptotic effects in all analyzed TNBC cell lines. Our results demonstrate that CAM and PAX used in combination act antagonistically, limiting anti-cancer efficacy and showing the importance of preclinical testing.

## 1. Introduction

Breast cancer (BC) is the most common malignancy in females worldwide [1]. Based on histopathological criteria including the expression of hormone receptors (estrogen receptor and/or progesterone receptor) and/or human epidermal growth factor receptor 2 (HER2), four main subtypes of BC were distinguished, including luminal A, luminal B, HER2 positive (HER2+) and basal-like or triple-negative breast cancer (TNBC) [2]. 

BC treatment includes surgical resection, radiotherapy, systemic chemotherapy, endocrine therapy and targeted therapy. Endocrine therapy has been shown to be effective in hormone receptor-positive BCs and is a common choice for adjuvant therapy. Yet, due to the aggressive nature of TNBC as well as lack of estrogen, progesterone and HER2 receptors, endocrine and targeted therapies are ineffective in the treatment of TNBC patients [2,3]. 

Chemotherapy, including anthracyclines (doxorubicin [4], epirubicin [4]), taxanes (paclitaxel [5], docetaxel [6]), platinum agents (cisplatin [7], carboplatin [8]) or 5-fluorouracil [9], is a first-line therapy of TNBC patients. However, chemoresistance for these drugs caused by changes in drug targets, drug inactivation, overexpression of ABC transporters, epithelial-mesenchymal transition (EMT), apoptotic dysregulation or cancer stem cells is a serious clinical problem that limits the effectiveness of chemotherapy [10]. Moreover, conventional chemotherapy is effective mainly in the initial stages; along with the progression of the disease, the efficacy of the therapy decreased and showed almost no effect in advanced stages of TNBC [3]. 

Recent insights into the TNBC complexity have been explained by epigenetic regulation and its ability to alter specific oncogenes and tumor suppressor genes. It has opened an emerging area in anti-cancer targeted therapy using epigenetic modulators [11]. The epigenetic drugs are synthetic or natural molecules capable of inhibiting or modulating the activity of epigenetic enzymes, e.g., DNA or histone methyltransferases, demethylases, histone acetyltransferases (HATs) or histone deacetylases (HDACs) [12]. 

HDACs are a large family of epigenetic metalloenzymes involved in gene transcription, cell differentiation, proliferation, migration, apoptosis or angiogenesis in cancer development and progression [13]. Sirtuins (SIRTs) belongings to IIIrd class of HDACs require NAD+ as a cofactor and include SIRT1-7 proteins in mammals [14]. Histone deacetylase inhibitors (HDIs), including sirtuin inhibitors (SIRTi), have been extensively studied for cancer treatment in the last decade. Despite the fact that they have shown high anti-cancer efficiency as well as tolerable side effects, their effectiveness in treating some types of solid tumors was insufficient. Nevertheless, several studies have demonstrated that drug efficacy problem can be overcome by the combined application of HDIs with other chemotherapeutic drugs [15,16].

**Figure 1:**
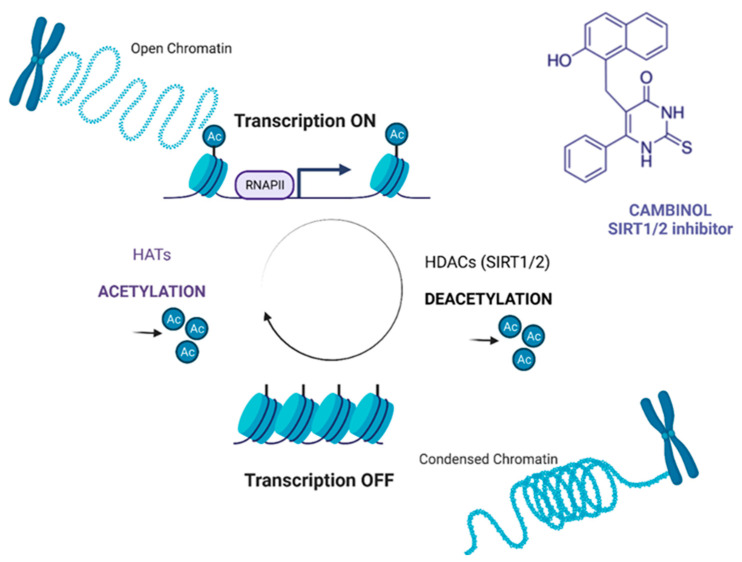
The mechanism of CAM action. CAM (SIRT 1/2 inhibitor) inhibits the activity of SIRT 1 and 2 (HDACs), which leads to the relaxation of chromatin and activation of transcription. HDACs–histone deacetylases, HATs–histone acetyltransferases, SIRT–sirtuin. The Figure was created with www.biorender.com (accessed on 22 May 2022).

Cambinol (SIRTi, HDIs) is a cell-permeable β-naphthol derivative that selectively inhibits NAD-dependent deacetylase activity of human SIRT1 and SIRT2. The molecular mechanism of CAM is still unclear; however, it has been demonstrated that CAM increases of p53, FOXO3a and Ku70 acetylation level through SIRT1 and SIRT2 inhibition, and as a consequence sensitizes NCI H460 lung cancer cells to paclitaxel, drug which is commonly used also in TNBC chemotherapy (Figure 1) [17].

Therefore, in our study, we determined a combined anti-cancer activity of CAM applied together with PAX in an experimental treatment against TNBC cells to determine their pharmacological drug-drug interaction trough the advanced isobolographic method. Moreover, we examined cytotoxic, anti-proliferative and pro-apoptotic effects of CAM used individually and combined with PAX in order to establish if this kind of treatment enhances the anti-proliferative and pro-apoptotic activity of CAM and PAX treatment in BT-549, MDA-MB-468 and HCC1937 TNBC cells. 

## 2. Results

### 2.1. CAM and PAX Administered Alone or Combined Decrease Viability of BT-549 and MDA-MB-468 TNBC Cells

**Figure 2:**
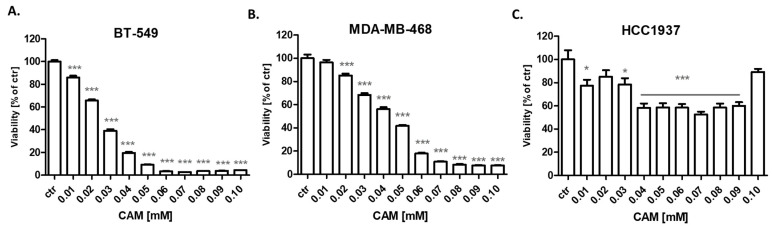
Viability of (**A**) BT-549 (**B**) MDA-MB-468 and (**C**) HCC1937 TNBC cells after 96 h incubation with 0.01–0.1 mM of CAM in the MTT assay. Statistical test: one-way ANOVA, Tukey post-hoc testing. * *p* < 0.05, *** *p* < 0.001. Data are presented as mean ± standard deviation (±SD).

**Figure 3:**
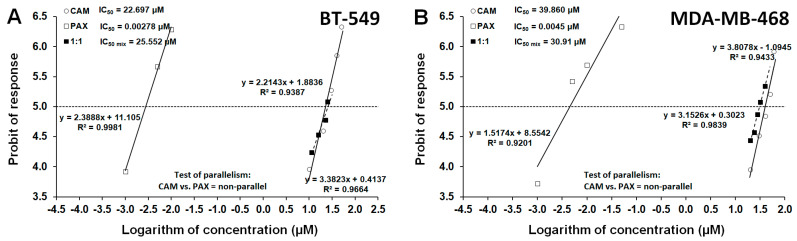
Log-probit concentration-effect relationship curves (CECs) for PAX and CAM used singly and in combination at the fixed-ratio of 1:1 for (**A**) BT-549 and (**B**) MDA-MB-468 TNBC cells. Concentrations of CAM and PAX were transformed into logarithms and the anti-proliferative effects into probits. The dotted line (parallel to the abscissa at 5th probit) indicates in approx. the IC_50_ values for PAX and CAM. Test of parallelism between PAX and CAM indicated that both lines are not collateral to each other.

The antiproliferative activity of CAM was assessed in a variety of TNBC cells using the MTT assay to establish IC_50_ values. The IC_50_ values were the concentrations resulting in 50% cell growth inhibition by a 96 h exposition to an active agent compared with control (untreated cells). BT-549, MDA-MB-468 and HCC1937 BC cell lines were exposed to either culture medium (control) or increasing concentrations of CAM (0.01–0.1 mM) (Figure 2). We have shown a decrease in the viability of BT-549 (Figure 2A) and MDA-MB-468 (Figure 2B) TNBC cells after CAM treatment in the dose-dependent fashion. CAM IC_50_ values for all investigated cell lines were established and depicted in Table 1. IC_50_ values for PAX were determined previously [18]. BT-549 was the most sensitive cell line to CAM with IC_50_ = 22.70 ± 2.23 μM; (Figure 3). In turn, treatment of HCC1937 BC cells with CAM did not allow the determination of the IC_50_ value (Figure 2C); therefore, only BT-549 and MDA-MB-468 TNBC lines were used to study the combined effects of CAM and PAX. Of note, this study was based on experimental evaluation of interaction between two drugs in mixture applied in a constant fixed-ratio combination of 1:1. In other words, all the studied drugs must exert the similar effect (i.e., inhibits cell proliferation in 50%, which corresponds in approx. to the IC_50_ value) so as to determine the effects exerted by the drugs in mixture. If one of the studied drugs is inactive in this experimental approach, the type II isobolographic analysis must be conducted [19,20]. This is the reason that only two cell lines (i.e., BT-549 and MDA-MB-468 TNBC) were used to determine the combined effects of CAM and PAX in this study. The test of parallelism is obligatory in combination experiments to properly classify interaction occurring between drugs [21,22].

The log-probit method to experimentally determine the IC_50_ values for the studied drugs (CAM and PAX when applied alone) and their combination (at the fixed-ratio of 1:1) in various cell lines was used, as recommended ealier [21,23]. Of note, the probit-type dose–response curves for CAM and PAX were verified in terms of their mutual parallelism based on the Loewe additivity model [21,24]. In such a situation, additivity was defined as the interaction if two drugs combined together exert the expected effects equal to the sum of effects produced by particular drugs when used separately. Sometimes this type of interaction was called “zero interaction” in contrast to antagonism (sub-additivity) or synergy (supra-additivity), when the observed effects were lesser or greater, respectively, than theoretically expected [21,24]. Log-probit analysis revealed that CAM had its dose–response line non-parallel to PAX in both, BT-549 and MDA-MB-468 TNBC lines (Figure 3). Of note, lack of parallelism between the analyzed lines forced us to perform type I isobolographic analysis for non-parallel concentration-response effect lines with additivity defined as an area bounded by two lower and upper isoboles of additivity [25,26]. 

### 2.2. CAM and PAX Administered Alone or Combined Decrease Proliferation of BT-549, MDA-MB-468 and HCC1937 TNBC Cells

**Figure 4:**
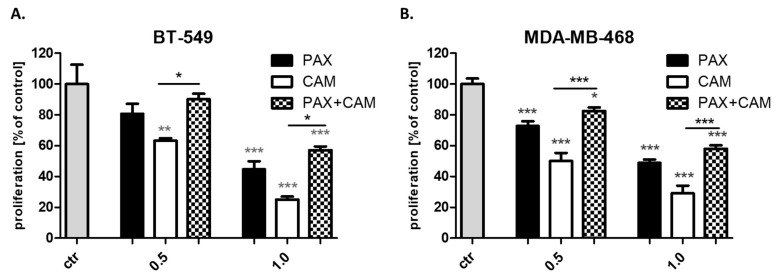
The proliferation of (**A**) BT-549 and (**B**) MDA-MB-468 TNBC cells in the BrdU assay after CAM and PAX treatment. BC cells were incubated for 48 h individually (control) or with the drugs (0.5 = 1/4 IC_50_; 1.0 = 1/2 IC_50_ determined in the MTT assay). Statistical test: one-way ANOVA, Tukey post-hoc testing. * *p* < 0.05, ** *p* < 0.01, *** *p* < 0.001. Data are presented as mean ± standard deviation of the mean (±SD). Grey *-data vs. ctr; black *-single treatment vs. combined treatment.

CAM and PAX administered alone dose-dependently reduce the proliferation of BT-549 and MDA-MB-468 TNBC cells as evaluated by measuring BrdU (5-bromo-2′deoxyuridine) incorporation into cellular DNA in proliferating cells (Figure 4). Treatment of all analyzed BC cells with CAM and PAX in combination (1:1 ratio) abolished the effect caused by these drugs used in monotherapy, proving that CAM and PAX act antagonistically.

### 2.3. Isobolographic Analysis of Drug-Drug Pharmacological Interaction between CAM and PAX for Non-Parallel Concentration-Effect Curves in BT-549 and MDA-MB-468 TNBC Cells

**Figure 5:**
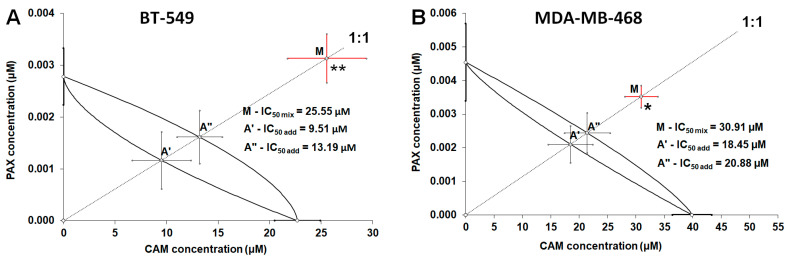
Isobolographic analysis of interactions between PAX and CAM in (**A**) BT-549 and (**B**) MDA-MB-468 TNBC lines measured in vitro by the MTT assay. The IC_50_ values for PAX and CAM are plotted on the ordinate and abscissa, respectively. The lower and upper isoboles of additivity represent the curves connecting the IC_50_ values for PAX and CAM. The points A′ and A″ depict the theoretically calculated IC_50 add_ values, whereas the point M represents the experimentally-derived IC_50 mix_ value. * *p* < 0.05 and ** *p* < 0.01 vs. the respective IC_50 add_ values (Student’s *t*-test with Welch correction).

With isobolographic analysis of interaction the mixture of PAX with CAM (at the fixed-ratio of 1:1) exerted sub-additive (antagonistic) interaction in the BT-549 TNBC cells (Table 2; Figure 5A). The experimentally derived IC_50 mix_ value for this fixed-ratio combination in the BT-549 cell line was 25.55 μM and considerably differed (t = 2.801; df = 155.8; *p* = 0.0057) from the additively calculated IC_50 add_ values, which were 9.51 μM (for the lower IC_50 add_) and 13.19 μM (for the upper IC_50 add_), respectively (Table 2, Figure 5A). Similarly, the mixture of PAX with CAM (1:1) exerted sub-additive (antagonistic) interaction in the MDA-MB-468 TNBC (Table 2; Figure 5B). Statistical analysis of data with Student’s *t*-test with Welch correction revealed that the IC_50 mix_ value (30.91 μM) significantly differed from the additively calculated IC_50 add_ values (18.45 μM-the lower IC_50 add_ and 20.88 μM-the upper IC_50 add_; t = 2.022; df = 275.3; *p* = 0.0441; Table 2, Figure 5B). 

### 2.4. CAM and PAX Administered Individually Induce Cell Death, While the Administration in Combi-Nation Abolishes This Effect in BT-549 and MDA-MB-468 TNBC Cells

**Figure 6:**
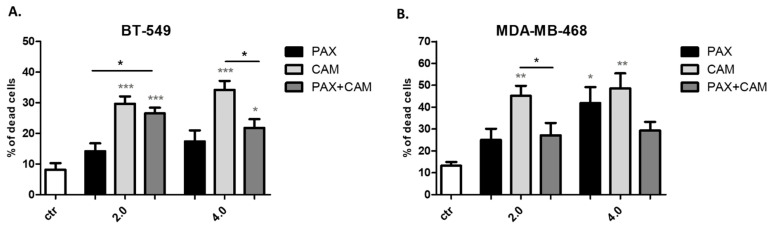
Effect of CAM and PAX alone or in combination on whole cell death induction in (**A**) BT-549 and (**B**) MDA-MB-468 TNBC cells in the trypan blue exclusion assay. BC cells were exposed to drugs treatment for 48h using selected ratios of the IC_50_ determined in the MTT assay (2.0 = IC_50_, 4.0 = 2IC_50_). Data are presented as mean ± standard deviation (±SD); one-way ANOVA, Tukey’s post-hoc testing; * *p* < 0.05, ** *p* < 0.01, *** *p* < 0.001. Grey *-data vs. ctr; black *-single treatment vs. combined treatment.

The effect of CAM and PAX applied individually or combined on the induction of whole-cell death was measured in the trypan blue exclusion assay (Figure 6). Both CAM and PAX used alone increased the dead cells number (blue-stained) in the dose-dependent fashion. This effect was much more evident in MDA-MB-468 cells than in BT-549 TNBC cells. The absence of significant synergistic cytotoxic effects between CAM and PAX determined in the isobolographic analyses was confirmed in both TNBC cell lines. 

### 2.5. CAM and PAX Administered Individually Induce Apoptosis, While the Administration in Combination Abolishes This Effect in BT-549 and MDA-MB-468 TNBC Cells 

**Figure 7:**
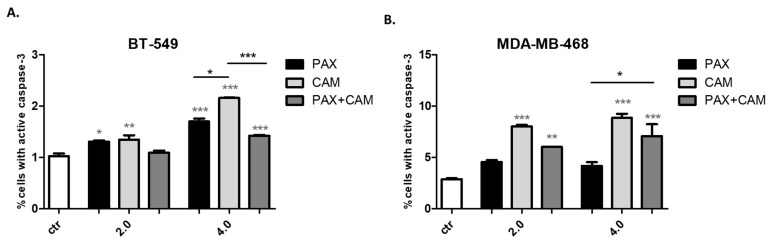
Effect of CAM and PAX alone or in combination on caspase-3 activation in (**A**) BT-549 and (**B**) MDA-MB-468 TNBC cells. BC cells were exposed to drugs treatment for 48h using selected ratios of the IC_50_ determined in the MTT assay (2.0 = IC_50_, 4.0 = 2IC_50_) and analyzed by FACS. Data are presented as mean ± standard deviation (±SD); one-way ANOVA, Tukey’s post-hoc testing; * *p* < 0.05, ** *p* < 0.01, *** *p* < 0.001. Grey *-data vs. ctr; black *-single treatment vs. combined treatment.

The influence of CAM and PAX administered separately or combined on induction of apoptosis was measured as a number of cells with activated caspase-3 (Figure 7). The appropriate concentrations of the drugs obtained from isobolographic analysis (IC_50_ and 2IC_50_) were used in the experiments. Both CAM and PAX used individually induced an increase in the number of cells with active caspase-3. Interestingly, CAM induced apoptosis more strongly than PAX in both analyzed cell lines (Figure 7). In MDA-MB-468 cells, the IC_50_ and 2IC_50_ decrease caused by PAX was insignificant compared to the control (Figure 7B). However, CAM and PAX used in combination significantly decreased the percentage of apoptotic cells, suggesting that PAX abolishes the effect of CAM. The difference in the level of active caspase-3 between control and CAM or PAX used alone or in combination was much weaker in BT-549 cells (Figure 7A) than in MDA-MB-468 cells (Figure 7B). Summarizing, the antagonistic pharmacological drug-drug interaction determined in isobolographic analysis was confirmed by assessing apoptosis for BT-549 and MDA-MB-468 TNBC cells.

**Figure 8:**
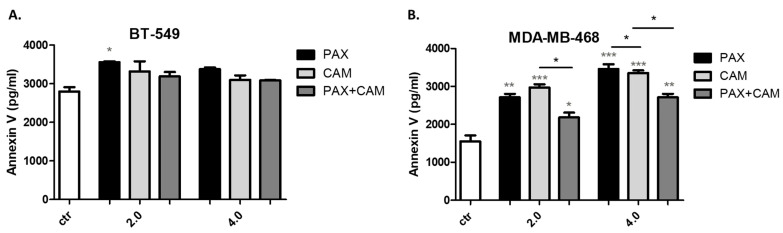
Interpolated protein concentration of Annexin V (pg/mL) in 100 µg/mL of protein, extracted from (**A**) BT-549 and (**B**) MDA-MB-468 TNBC cells after CAM and PAX treatment, used individually or in combination. BC cells were exposed to drugs treatment for 48h using selected ratios of the IC_50_ determined in the MTT assay (2.0 = IC_50_, 4.0 = 2IC_50_). Concentrations of Annexin V were measured in triplicate and interpolated from the Annexin V standard curve and corrected for sample dilution. The interpolated dilution factor corrected values are plotted (mean ± SD, n = 3).; one-way ANOVA, Tukey’s post-hoc testing; * *p* < 0.05, ** *p* < 0.01, *** *p* < 0.001. Grey *-data vs. ctr; black *-single treatment vs. combined treatment.

Annexin V concentrations (pg/mL) in 100 µg/mL of protein extracted from BT-549 and MDA-MB-468 TNBC cells were determined using Human Annexin V Elisa Assay and demonstrated in Figure 8. Concentration of Annexin V was higher in BT-549 (2802 ± 110 pg/mL) than in MDA-MB-468 (1553 ± 155.4 pg/mL) control TNBC cells. CAM and PAX used alone induced a protein expression of Annexin V in both analyzed TNBC lines; however, changes were statistically insignificant for the BT-549 BC cell line. Interestingly, a combination of CAM and PAX abolishes this effect, confirming the PAX:CAM antagonistic interaction.

### 2.6. CAM and PAX Administered Singly and in Combination Induce Cell Cycle Arrest in the BT-549 and MDA-MB-468 BC Cells in a Cell-Dependent Manner

**Figure 9:**
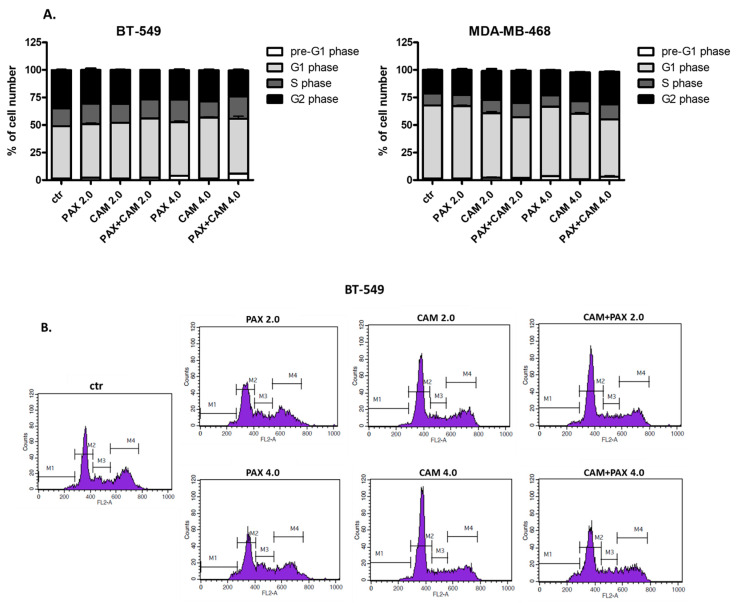
(**A**) Influence of CAM and PAX on cell cycle progression in the BT-549 and MDA-MB-468 TNBC cells. BC cells were exposed to an individual or concomitant CAM and PAX treatment for 48 h using selected ratios (2.0 = IC_50_, 4.0 = 2IC_50_) of the IC_50_ determined in the MTT assay, stained with propidium iodide (PI) and analyzed by FACS. The data are presented as the means ± standard deviation (±SD). (**B**) Representative histograms from the FACS analysis of the BT-549 TNBC cells after a 48 h incubation with a medium (ctr), PAX, CAM and PAX + CAM (2.0 = IC_50_, 4.0 = 2IC_50_). M1-pre-G1 phase; M2-G1 phase; M3-S phase; M4-G2 phase.

The effect of CAM and PAX treatment (individually or in combination) on the cell cycle progression was examined in BT-549 (Figure 9A,B) and MDA-MB-468 (Figure 9A) TNBC cells (Table 3). FACS analysis of PI-stained cells demonstrated that incubation of both analyzed BC cells with PAX for 48h led to the accumulation of cells in the pre-G1 phase in the dose-dependent manner (Figure 9A,B). This effect was more pronounced in BT-549 cells comparing MDA-MB-468 cell line. Incubation with CAM resulted in an increase of cell numbers in the G2 phase, followed by a reduction of cell numbers in the G1 phase in MDA-MB-468 BC cells. The opposite effect was observed in the BT-549 cell line (an increase of cell number in G1 and decrease in G2 phases). Simultaneous exposure to CAM and PAX maintained PAX induced pre-G1 arrest in both cell lines. Incubation of BT-549 and MDA-MB-468 BC cells with CAM and PAX resulted in a cell cycle inhibition pattern similar to that induced by PAX alone (Figure 9A,B).

## 3. Discussion

Standard methods of TNBC treatment include chemotherapy with platinum agents [7,8], anthracyclines [4], 5-fluorouracil [9] or taxanes [5,6]. The efficiency of PAX is limited due to its cytotoxicity to normal cells and consequently the development of several adverse effects as well as PAX resistance [5,6]. Thus, on the one hand, new, more effective chemotherapeutic drugs which selectively eliminate BC cells are being looked for. On the other hand, combinations of established anti-cancer drugs and new cytostatics are being tested in order to enhance the anti-cancer properties of currently used chemotherapeutics without destroying healthy tissue. In this context, a broad range of both natural as well as synthetic compounds, including HDIs, have been identified and have become an interesting class of active agents in the therapy of TNBC. HDIs, including SIRTi are promising anti-cancer drugs due to their ability to induce apoptosis or inhibit proliferation and cell cycle in numerous cancer cells [2,27,28], including BC [29]. Although in clinical trials, HDIs did not demonstrate spectacular therapeutic effects in BC treatment as single agents, it has been shown that HDIs increase the sensitivity of BC cells to radio- and chemotherapy [27,28]. CAM is the selective SIRT1 and SIRT2 (3rd class of HDACs) inhibitor. The molecular mechanism of CAM is still unclear; however, it has been shown that CAM increases p53, FOXO3a and Ku70 acetylation level through SIRT1 and SIRT2 inhibition and consequently sensitizes cancer cells (e.g., NCI H460 lung cancer cells) to PAX, which is ordinarily also utilized likewise in TNBC therapy [17]. Moreover, it has been demonstrated that SIRT1/2 inhibition caused by CAM perturbs Frizzled 7 (FZD7) mRNA and protein levels, suggesting that sirtuins may play a regulatory role in FZD receptor expression [30]. However, there are no in vivo studies and clinical trials on CAM activity in BC patients. 

Therefore, in our manuscript, we have tested the cytotoxic, anti-proliferative and pro-apoptotic activity of CAM used individually or in combination with PAX in different TNBC cell lines. Moreover, we have determined types of pharmacological CAM:PAX interactions in vitro using the isobolographic method in BT-549 and MDA-MB-468 BC cells in order to check if this combination is more beneficial in TNBC than single agents therapy. Our studies have shown a dose-dependent decrease in the viability and proliferation of BT-549 and MDA-MB-468 cells after CAM treatment. These results remain in line with previously reported findings for neuroblastoma [31], hepatocellular carcinoma [32] or pancreatic cancer cells in vitro [33] as well as Burkitt lymphoma mouse xenograft model [34]. However, treatment of all analyzed BC cells with CAM and PAX in combination abolished the anti-proliferative effect caused by these drugs used in monotherapy, proving that CAM and PAX act antagonistically. Similarly to cytotoxic and anti-proliferative studies, CAM and PAX used alone induced an increase in the number of apoptotic cells. In turn, CAM and PAX in combination significantly decreased the percentage of caspase-3-positive cells, suggesting that PAX overthrows the effect of CAM. Antagonistic pharmacological CAM:PAX interaction we were confirmed in isobolographic analysis for BT-549 and MDA-MB-468 TNBC cells. Similar results were obtained in our previous study [35]. CAM and another chemotherapeutic-CDDP administered separately induced inhibition of cell viability in each analyzed BC cell line in a dose-dependent manner. However, CAM in combination with CDDP significantly decreased cytotoxicity and the percentage of cells with active caspase-3 caused by these drugs used alone. An isobolographic analysis confirmed the antagonistic effect of CDDP and CAM in BC cell lines with different phenotypes: MCF7, T47D estrogen receptor-positive and MDA-MB-231, MDA-MB-468 TNBC cells [35]. Additionally, CAM in combination with doxorubicin did not show a significant synergetic effect in neuroblastoma cells; although, CAM applied individually was effective in DOX-sensitive cells [36].

To conclude, our data indicate that CAM and PAX used in combination produce antagonistic interaction. The pharmacological drug–drug interaction between CAM and PAX disclaims the potential application of combined treatment in TNBC cells. However, CAM used individually demonstrates strong chemotherapeutic potential; therefore, it should be tested in co-therapy with other chemotherapeutics with different molecular mechanisms of action than PAX.

## 4. Materials and Methods

### 4.1. Cell Culture 

Human BT-549, MDA-MB-468 and HCC1937 TNBC cells were obtained from the American Type Culture Collection (ATTC). Mycoplasma free cells were maintained in a 1:1 mixture of Dulbecco’s modified Eagle’s medium (DMEM) and HAM’s F12 (Sigma, St. Louis, MO, USA) supplemented with 10% *v*/*v* fetal bovine serum (FBS) and antibiotics: penicillin (100 IU/mL) and streptomycin (100 µg/mL) (Sigma). The cultures were maintained at 37 °C in a humidified atmosphere containing 5% CO_2_.

### 4.2. Cells Treatment

CAM (Sigma) and PAX (Sigma) were dissolved in dimethyl sulfoxide (DMSO) (Sigma) at 1 mM and 10 mM, respectively. In order to obtain the final concentrations, stock solutions were diluted in the DMEM/HAM’s F12 culture medium just before use. Cells at an optimized density of 1.0 × 10^4^ cells/mL were incubated for 96 h with increasing concentrations of the drugs to determine the IC_50_ concentration for each compound. Typically, cells were equally seeded and kept under standard growing conditions for 24 h. The following day, CAM and PAX were supplemented to fresh media, either individually or in combination, and TNBC cells were incubated for times and concentrations provided in each experimental condition; 1.0 = ½ IC_50_, e.g., for BT-549 C_PAX_ = 0.00278 µM, C_CAM_ = 22.696 µM (individual treatment), C_PAX + CAM_ = 0.00139 µM _PAX_ + 11.348 µM _CAM_ (combined treatment).

### 4.3. Cell Viability Assay (MTT)

Inhibition of cancer cells viability was evaluated by 3-(4,5-dimethylthiazol-2-yl)-2,5-diphenyltetrazolium bromide (MTT) assay as we described previously [29]. BT-549, MDA-MB-468 and HCC1937 TNBC cells (1 × 10^4^ cells/mL) were treated with CAM (0.01–0.1 mM) and PAX (0.001–1 µM) individually or in combination for 96 h. After treatment with the examined compounds the cells were incubated with 10 μL of MTT solution (5 mg/mL, Sigma). The reaction was stopped using 10% SDS in 0.01N HCl solution. Finally, absorbance was measured at 570 nm (Infinite M200 Pro Microplate Reader, Tecan, Männedorf, Switzerland). 

### 4.4. Cell Proliferation Assay (ELISA BrdU)

Inhibition of cancer cells proliferation was determined using Cell Proliferation Elisa, BrdU Kit (Roche, Germany; Cat. No. 11647229001) as we described previously [37]. Optimized density (1 × 10^4^ cells/mL) of BT-549 and MDA-MB-468 TNBC cells were placed on a 96-well plate (Nunc, Thermo Fisher Scientific, CA, US) and then treated with selected concentrations (1/2 IC_50_ and IC_50_) of PAX and CAM individually or in combination for 48 h, followed by incubation with 5-bromo-2-deoxyuridine (BrdU) at 100 μM. Quantitation of final product of the enzymatic reaction was performed spectrophotometrically at 450 nm using the Infinite M200 Pro microplate reader (Tecan).

### 4.5. Isobolographic Analysis 

Pharmacodynamic interactions between PAX and CAM were classified by using the type I isobolographic analysis for non-parallel CECs in two TNBC cell lines, as described in details earlier [26,29]. With quantal log-probit analysis we determined CECs for PAX and CAM, when administered alone and in combination at the fixed-ratio of 1:1. In this study, percentage of the inhibition of cell viability was transformed to probit (in BT-549 and MDA-MB-468 TNBC cells measured by the MTT assay). The test of parallelism between concentration-effect curves of PAX and CAM was performed as described in details earlier [18,29]. Since the concentration-effect curves for PAX and CAM were non-parallel to one another, we calculated two IC_50 add_ values for the lower and upper isoboles of additivity, as presented earlier [23]. We observed sub-additivity (antagonism) in BT-549 and MDA-MB-468 TNBC cell lines because the IC_50 mix_ values were placed significantly above the area bounded by the upper isobole of additivity. After calculating the additive values (IC_50 add_) from the equation of additivity (based on Loewe’s additivity and zero interaction model) for the studied combination [21,38], we statistically compared the experimentally determined IC_50_ mix values with their respective IC_50 add_ values by means of the parametric test, as recommended elsewhere [39]. To statistically compare whether the studied two-drug combination produce synergy, additivity or antagonism, we analyzed data with Student’s *t*-test with Welch correction. If the statistically compared IC_50_ values considerably differ, synergy or antagonism between the tested drugs occurs. Synergistic interaction is observed if the IC_50 mix_ is significantly lower than the IC_50 add_. Antagonism occurs if the IC_50 mix_ is significantly higher than the IC_50 add_. In contrast, if both IC_50 mix_ and IC_50 add_ values do not differ, we are obliged to accept that the tested combination exerts additive interaction [23]. More detailed information about isobolographic analysis accompanied with equations on how to calculate additive values has been presented elsewhere [25]. 

### 4.6. Trypan Blue Exclusion Assay

BT-549 and MDA-MB-468 TNBC cells (1 × 10^5^) were moved in 6-well plates (Nunc) and kept in a standard growing medium for 24 h. Then, CAM and PAX, either alone or in combination, were subsequently added to new media and were incubated for 48 h. The pelleted cells were resuspended in 2 mL of DMEM/HAM F12 medium and diluted 1:1 with trypan blue, which, crossing the damaged membrane, discriminates living cells from dead cells. Next, 10 μL of both media containing cells and blue dye (0.4%, *v*/*v*) were mixed, and the number of unstained (living) and stained (dead) cells was recorded using an automatic TC20 BioRad Automated Cell Counter (Herkules, CA, USA). Each point has been counted at least twice in each experimental procedure.

### 4.7. Assessment of Apoptosis (FACS Analysis) 

BT-549 and MDA-MB-468 TNBC cell lines were seeded on 6-well plates at a density of 0.5 × 10^5^/mL and treated with CAM and PAX alone or in combination for 48 h. The measurement of apoptosis was conducted according to the manufacturer’s protocol of PE Active Caspase-3 Apoptosis Kit (BD Pharmingen, NJ, USA; Cat. No. 550914) as described previously [29]. 

### 4.8. Annexin V ELISA Assay

The quantitative measurement of the Annexin V protein in the BT-549 and MDA-MB-468 TNBC cells after CAM and PAX treatment was performed using a Human Annexin V ELISA^®^ Kit; Abcam (Cambridge, UK; Cat. No. ab119503). To perform the assay, the samples and the standards were added to the wells, followed by the antibody mix. After 1 h incubation at room temperature, the wells were washed with 350 µL wash buffer to remove the unbound material. Then, 100 µL of the TMB development solution was added to each well and incubated for 10 min. The color reaction was stopped by adding 100 µL of a stop solution. Finally, the intensity of the product was measured spectrophotometrically at 450 nm.

### 4.9. Cell Cycle Progression (FACS Analysis)

To determine cell cycle distribution, BT-549 and MDA-MB-468 TNBC cells were seeded on 6-well plates (Nunc) at a density of 1 × 10^6^ cells/mL. Next day the cells were treated with different concentrations of CAM and PAX alone or in combination (CAM:PAX) for 48 h and then fixed in ice-cold 80% ethanol at −20 °C. After fixation, the treated cells were stained with propidium iodide (PI) utilizing the PI/RNase Staining Buffer (BD Biosciences, NJ, USA; Cat. No. 550825) according to the manufacturer’s protocol. Experiments were performed using the FACSCalibur^TM^ flow cytometer (BD Biosciences). Acquisition rate was at least 60 events per second in low acquisition mode and at least 10,000 events were measured. Cell cycle analysis was performed by using flow cytometry analyzing software-Cylchred Version 1.0.2 for Windows (source: University of Wales) and WinMDI 2.9 for Windows (source: facs.scripps.edu/software.html). The cells were acquired and gated by using dot plot FL-2 width (x) versus FL-2 area (y)-gate to exclude aggregates and analyzed in histograms displaying fluorescence 2-area (yellow-orange fluorescence: 585 nm).

### 4.10. Statistics

GraphPad Prism 5.0 software was used for data analysis (one-way ANOVA with Tukey’s post-hoc testing). *p* < 0.05 was considered to indicate a statistically significant difference (* *p* < 0.05, ** *p* < 0.01, *** *p* < 0.001). Results were presented as mean±standard deviation of the mean (±SD) from 3 independent experiments. The IC_50_ and IC_50 mix_ values for PAX and CAM were calculated by quantal log-probit analysis, as reported earlier [29]. The experimentally-derived IC_50 mix_ values for the mixture of PAX and CAM were statistically compared with the theoretically additive IC_50 add_ values with unpaired Student’s *t*-test with Welch correction, as reported earlier [23]. 

## Figures and Tables

**Table 1:** Median inhibitory concentrations (IC_50_ ± SEM) for the PAX [18] and CAM in BT-549, MDA-MB-468 and HCC1937 TNBC cells. The IC_50_ values (in μM ± SEM) for PAX and CAM, when administered separately, were determined with log-probit method in three TNBC cells (BT-549, MDA-MB-468 and HCC1937) measured in vitro by the MTT assay. *n*–number of items; S–slope function ratio; f ratio; S–factor for slope function ratio; N.D.–not determined; NP—not parallel.

Cell Line	Drug	IC_50_ (μM)	*n*	S	f Ratio S	Parallelism
BT-549	PAX	0.00278 ± 0.00055 [18]	48	1.327	1.220	NP
	CAM	22.696 ± 2.227	96			
MDA-MB-468	PAX	0.00454 ± 0.00115 [18]	72	2.491	1.265	NP
	CAM	39.860 ± 3.475	96			
HCC1937	PAX	0.0186 ± 0.0084 [18]	72	N.D.	N.D.	N.D.
	CAM	N.D.	N.D.			

**Table 2:** Isobolographic analysis of interactions between PAX and CAM in BT-548 and MDA-MB-468 TNBC cells. The IC_50_ values (in μM ± SEM) for two-drug mixtures were determined experimentally (IC_50 mix_) and theoretically calculated (IC_50 add_) from the equations of additivity in the BT-548 and MDA-MB-468 TNBC cell lines measured in vitro by the MTT assay. *n* _mix_–total number of items for the experimental mixture; *n* _add_–total number of items for the additive mixture; * *p* < 0.05 and ** *p* < 0.01 vs. the respective IC_50 add_ values (Student’s *t*-test with Welch correction).

Cell Line	IC_50 mix_ (μM)	n_mix_	Lower-IC_50 add_ (μM)	n_add_	Upper-IC_50 add_ (μM)	Interaction
BT-548	25.55 ± 3.83 **	96	9.51 ± 2.19	140	13.19 ± 2.86	Antagonism
MDA-MB-468	30.91 ± 2.91*	120	18.45 ± 3.91	164	20.88 ± 4.01	Antagonism

**Table 3:** Cell cycle distribution of BT-549 and MDA-MB-468 TNBC cells exposed to an individual or concomitant CAM and PAX treatment for 48 h using selected ratios (2.0 = IC_50_, 4.0 = 2IC_50_) of the IC_50_ determined in the MTT assay, stained with propidium iodide (PI) and analyzed by FACS. The data in the table are presented as the means ± standard deviation (±SD) of the means.

Cell Line	Drug	Pre-G1 Phase [%]	G1 Phase [%]	S Phase [%]	G2 Phase [%]
BT-549	Control	1.2780 ± 0.38960	47.5600 ± 0.3742	16.4300 ± 0.5751	34.590 ± 0.7851
	PAX 2.0	2.1380 ± 0.32830	48.8100 ± 1.7220	18.5200 ± 0.9863	30.510 ± 2.9130
	CAM 2.0	1.4680 ± 0.20320	50.6000 ± 0.8995	17.1700 ± 0.8412	30.790 ± 0.4281
	PAX + CAM 2.0	2.3450 ± 0.10470	53.6100 ± 0.5734	17.3200 ± 0.5867	26.630 ± 0.4492
	PAX 4.0	4.0600 ± 0.33380	48.4300 ± 2.7960	20.4600 ± 1.0240	26.920 ± 1.6980
	CAM 4.0	1.2830 ± 0.12090	55.5900 ± 1.0830	14.6100 ± 0.2636	28.370 ± 0.8651
	PAX + CAM 4.0	5.8450 ± 0.62700	49.7600 ± 4.9960	20.3900 ± 2.8960	23.480 ± 1.5260
MDA-MB-468	Control	1.5550 ± 0.24030	66.1400 ± 0.9984	10.8500 ± 0.3469	21.410 ± 1.0550
	PAX 2.0	1.4830 ± 0.43570	65.7800 ± 1.6210	10.0200 ± 0.2220	22.690 ± 1.8650
	CAM 2.0	2.1150 ± 1.59600	58.5100 ± 3.2190	12.2600 ± 0.2891	26.200 ± 3.5080
	PAX + CAM 2.0	1.9030 ± 0.43650	55.0600 ± 1.1350	13.0600 ± 0.4994	29.330 ± 1.6970
	PAX 4.0	3.4900 ± 0.15100	63.2700 ± 0.3439	10.3300 ± 0.4325	22.720 ± 0.2868
	CAM 4.0	0.7800 ± 0.03162	59.3000 ± 2.7610	11.5300 ± 1.7360	26.370 ± 0.4074
	PAX + CAM 4.0	3.0900 ± 1.74900	51.8900 ± 1.2350	13.9600 ± 0.7807	29.440 ± 0.6453

## Data Availability

Not applicable.
